# Differences in Switching Away From Smoking Among Adult Smokers Using JUUL Products in Regions With Different Maximum Nicotine Concentrations: North America and the United Kingdom

**DOI:** 10.1093/ntr/ntab062

**Published:** 2021-05-18

**Authors:** Nicholas I Goldenson, Yu Ding, Shivaani Prakash, Cameron Hatcher, Erik M Augustson, Saul Shiffman

**Affiliations:** 1 Juul Labs, Inc., Washington, DC, USA; 2 PinneyAssociates, Inc., Bethesda, MD, USA

## Abstract

**Introduction:**

Electronic nicotine delivery systems (ENDS) may improve public health if they facilitate smokers switching away from cigarettes. Conceptually, switching is facilitated when ENDS provide adequate nicotine delivery. Switching rates among smokers who purchased the JUUL System (“JUUL”) were compared in the United Kingdom (UK), where regulations limit nicotine concentration to 20 mg/mL versus North America (N.Am.; United States and Canada), where higher concentrations are available.

**Aims and Methods:**

Adult established smokers (age ≥21, smoked ≥100 cigarettes, smoking some days or every day at baseline) who newly purchased JUUL were recruited into a longitudinal study (UK: *N* = 1247; N.Am.: *N* = 8835). Complete switching (no smoking for ≥30 days) was assessed 1, 3, and 6 months after purchase; propensity score matching (PSM) and logistic regression compared switching after adjusting for baseline characteristics.

**Results:**

In both N.Am. and UK, ≥82% of participants reported using the highest JUUL nicotine concentration available (UK: 18 mg/mL; N.Am.: 59 mg/mL). Unadjusted switching rates did not differ at 1 month (17%–18%); unadjusted and adjusted rates were significantly higher in N.Am. (vs. UK) at 3 and 6 months. In the PSM sample, after additional covariate adjustment, rates were significantly higher in N.Am. (vs. UK) at 3 months (31.5% vs. 22.7%; odds ratio [95% confidence interval, CI] = 1.59 [1.25, 2.02]) and 6 months (38.0% vs. 26.0%; odds ratio [95% CI] = 1.79 [1.37, 2.35]).

**Conclusions:**

These results suggest availability of ENDS in nicotine concentrations greater than 20 mg/mL may be associated with increased switching among adult smokers. Differences in smoking and ENDS use characteristics did not explain associations of location and switching; however, between-country differences may be affected by unmeasured factors.

**Implications:**

Switching rates were lower among smokers who purchased the JUUL System (“JUUL”) in the UK, where regulations limit nicotine concentration to 20 mg/mL versus N.Am. (United States and Canada), where higher concentrations are available—before and after controlling for differences in smoking and ENDS use characteristics. These results suggest availability of ENDS in nicotine concentrations greater than 20 mg/mL may be associated with increased switching among adult smokers. Between-country differences may be affected by unmeasured factors; future research should consider these factors and the extent to which regulatory policy environments may explain differences in switching among adult smokers.

## Introduction

Cigarette smoking remains the leading cause of preventable morbidity and mortality worldwide.^1^ Smoking is persistent, with very low likelihood of cessation for any given quit attempt.^2^ Nicotine is the primary constituent that maintains cigarette smoking.^3–5^ However, nicotine itself is not the major source of the harms of smoking.^1,6^ As Russell aptly stated decades ago, “People smoke for nicotine but they die from the tar.”  ^7^

Tobacco harm reduction involves transitioning smokers who would not otherwise quit smoking to noncombustible nicotine delivery products, decreasing their exposure to harmful constituents.^6,8^ Electronic nicotine delivery systems (ENDS) are representative of this strategy.^9^ Although ENDS are not positioned or approved as medications for smoking cessation, findings from randomized cessation trials suggest that ENDS can help smokers quit smoking as well as or better than nicotine replacement medications.^10,11^

Consistent with the conceptualization of ENDS as consumer products intended to draw smokers away from cigarettes, observational evidence demonstrates that many smokers who adopt ENDS are able to switch completely away from smoking.^11^ Two studies found that substantial proportions of US adult smokers who purchased a JUUL Starter Kit (henceforth “JUUL”) reported switching away from smoking (ie, no past 30-day smoking) 3 and 6 months later.^12,13^ However, the JUUL products in those studies contained 5.0% nicotine by weight (59 mg/mL), and it is unknown whether switch rates would be affected with lower nicotine concentrations.

Conceptually, switching is expected to be facilitated when ENDS provide adequate nicotine delivery,^6,14,15^ as dose–response effects are fundamental to pharmacological action of nicotine.^4,16^ In the context of nicotine replacement, randomized clinical trials demonstrate that nicotine gum with higher doses is more effective in heavier and more dependent smokers.^17^ A 2013 cross-sectional survey found that use of ENDS with higher nicotine concentrations was associated with increased switching, and that over 15% of smokers increased nicotine levels in their ENDS to switch.^18^ However, there is a lack of recent and longer-term longitudinal data assessing differences in switching away from smoking by ENDS nicotine concentration.

Differences across regulatory settings provide data to address this question. Unlike the United States and Canada (North America [N.Am.]), the United Kingdom (UK) limits the maximum nicotine concentration for ENDS to 20 mg/mL (via Tobacco Products Directive [TPD]).^19^ This natural variation across policy regions provides an opportunity to assess the effect of nicotine concentration on the likelihood of switching among smokers. A recent analysis evaluated changes in smoking and ENDS use in the United States, Canada, and England over 18 months, but did not assess nicotine concentration or parse heterogeneity in ENDS products within and across countries.^20^

The current study compared switching rates with JUUL in the UK, where JUUL is available only in 18 and 9 mg/mL nicotine concentrations, to N.Am., where JUUL is available in 59 and 35 mg/mL. We used propensity score matching (PSM) to account for cross-country differences as reflected in individual profiles of smokers in each region. Countries may also differ in other ways (eg, smoking culture, tobacco control policies), so we also assessed the potential effects of unmeasured confounding.

## Methods

### Participants

Smokers in the United States, Canada, and UK were enrolled in three parallel longitudinal studies that assessed switching following their purchase of JUUL in a retail store or online (manufacturer’s ecommerce platform). Data were collected January 2019 to December 2020. Eligibility criteria for the analyses were: (1) of legal age to purchase JUUL; (2) permanent resident of the relevant country; and (3) purchase of a JUUL Device Kit or JUUL Starter Kit for the first time within 3 days prior to completing the baseline assessment. Employees of Juul Labs, Inc or PAX Labs, Inc and their relatives were ineligible. The analytic sample included only baseline established smokers (smoked ≥100 cigarettes, smoked in past 30 days, currently smoke “some days” or “every day”) over the age of 21 (UK participants could enroll at age 18; *N* = 387 UK participants under age 21 were excluded to make the groups comparable; Supplementary Figure S1). N.Am. participants (US and Canadian) were combined.

### Procedure

Individuals who purchased a JUUL Starter Kit or Device Kit directly from retail stores (via recruitment card in packaging) or online (via post-purchase email) were invited to participate (“Complete our online survey about vaping, smoking, and JUUL products”). Invitation cards were inserted into the packaging of JUUL Device Kits and JUUL Starter Kits distributed at random to licensed retail stores. After completing the baseline survey, participants received email invitations to complete the 1-, 3-, and 6-month follow-ups, online in English. Data were collected by the Centre for Substance Use Research (CSUR; Glasgow, Scotland; www.csures.com). The Advarra IRB approved the study protocol for United States and Canada; no ethics approval was required for the UK per the National Health Service Health Research Authority Governance Arrangements for Research Ethics Committees. All participants provided informed consent electronically and were compensated for each survey they completed (United States: $30; UK: £25; Canada: $40 CAD).

### Measures

#### Past 30-Day “Switching” Away From Smoking

At each follow-up participants who reported that they had not smoked in the past 30 days (“even one or two puffs”) were considered to have switched.

#### Primary JUUL Nicotine Concentration Used in the Past 30 Days

At each follow-up participants reported the total number of JUULpods they used in each nicotine concentration in the past 30 days. Participants’ primary JUUL nicotine concentration was operationalized as the nicotine concentration for which they used greatest number of pods in the past 30 days. Canadian participants who primarily used 1.5% nicotine concentration (18 mg/mL; available in Canada but not the United States) were excluded at each follow-up (*n* = 61, 59, and 59, at months 1, 3, and 6, respectively; *n* = 5 at all three timepoints) to isolate the effects of nicotine concentration.

### Covariates

Participant-level factors associated with switching in the smoking cessation and ENDS literatures were included as a priori covariates.^21–26^ Participants reported their age, sex, race/ethnicity (coded as non-Hispanic White vs. non-White), and marital status (Table 1). Assessed smoking characteristics included age started smoking regularly (continuous), daily cigarette consumption (number of days smoked cigarettes in past 30 days × number of cigarettes/day]/30) and baseline cigarette dependence (assessed with the 4-item Patient-Reported Outcomes Measurement Information System [PROMIS] Nicotine Dependence scale [range: 0–4^27,28]^). Participants also reported relative harm perceptions for JUUL (“In your opinion, is using the JUUL device likely to be less harmful, about the same, or more harmful to your health compared with smoking cigarettes?”) and reasons for JUUL use (advised by doctor, to help quit smoking, less harmful than smoking [select-all-that-apply]).

**Table 1.** Baseline Sociodemographic, Smoking and JUUL Use Characteristics, and Primary JUUL Nicotine Concentration at Follow-up by Nicotine Concentration Policy Region

	North America (*N* = 8835)	United Kingdom (*N* = 1247)	Difference (*p*)^a^
Sociodemographic characteristics			
Age, yr, mean (SD)	37.99 (11.96)	33.50 (10.95)	<.001
Sex			
Male	4677 (52.9)	824 (66.1)	<.001
Female	4122 (46.7)	420 (33.7)	
Transgender	36 (0.4)	3 (0.2)	
Non-Hispanic White Race (vs. Other Race)	6739 (76.4)	1020 (82.5)	<.001
Marital status			
Married	3308 (37.4)	295 (23.7)	<.001
Divorced, separated, or widowed	1610 (18.2)	129 (10.3)	
Never married	3876 (43.9)	799 (64.1)	
Smoking characteristics			
No. days smoked in past 30 days, mean (SD)	25.02 (8.62)	23.10 (9.74)	<.001
No. cigarettes smoked per day, mean (SD)	13.19 (12.33)	11.13 (10.44)	<.001
Age started smoking regularly, yr, mean (SD)	18.19 (4.16)	17.92 (3.34)	.03
Cigarette dependence,^b^ mean (SD)	2.10 (0.98)	1.93 (0.95)	<.001
JUUL use characteristics			
Relative harm of JUUL vs. cigarettes^c^			
Much less harmful	1838 (20.8)	410 (32.9)	<.001
Less harmful	5101 (57.7)	717 (57.5)	
About the same level of harm	1119 (12.7)	58 (4.7)	
More harmful	58 (0.7)	1 (0.1)	
Much more harmful	36 (0.4)	4 (0.3)	
I don’t know	683 (7.7)	57 (4.6)	
Reasons for JUUL use^d^			
Doctor advice	234 (2.7)	58 (4.7)	<.001
To help to quit smoking	6849 (77.5)	912 (73.1)	<.001
Healthier alternative to cigarettes	4800 (54.3)	769 (61.7)	<.001
Primary use of JUUL in highest available nicotine concentration^e^			
1-Month follow-up	6151 (92.5)	832 (90.1)	.01
3-Month follow-up	4868 (88.7)	611 (84.6)	.001
6-Month follow-up	3819 (89.1)	414 (82.3)	<.001

Values represent *N* (%) unless noted otherwise. Sample sizes or denominators may be less than column heads due to missing data.

^a^Differences between nicotine concentration policy regions were tested with χ  ^2^ for categorical variables and one-way analysis of variance for continuous variables.

^b^PROMIS cigarette dependence (range: 0–4).

^c^“In your opinion, is using the JUUL device likely to be less harmful, about the same, or more harmful to your health compared to smoking cigarettes?.”

^d^Respondents were allowed to choose multiple reasons for JUUL use (select-all-that-apply format).

^e^Nicotine concentration used most often in the past 30 days (concentration with greatest number of pods used in past 30 days); North America: 59 mg/mL (vs. 35 mg/mL); UK: 18 mg/mL (vs. 9 mg/mL). Users without a primary concentration were excluded.

#### Past 30-Day JUUL Use Across Follow-up

Past 30-day JUUL use (yes/no) was assessed at each follow-up.

### Statistical Analysis

Initial analyses tested differences in demographic and smoking-history variables by nicotine concentration policy region (N.Am. vs. UK). Differences in switching rates were assessed separately at 1, 3, and 6 months, as respondents’ switching status could change at each follow-up, and some participants were missing switching data at some follow-ups.

Differences in switching at each follow-up between N.Am. and UK were assessed in four sets of logistic regression models. First, models assessed switching rates in the full (unmatched) sample, without and with adjustment for all a priori covariates. Then, PSM was used to create matched samples of N.Am. and UK smokers that were similar on observed covariates; differences in switching were tested without and with baseline covariate adjustment. The average marginal effects from adjusted logistic regression models were used to calculate the covariate-adjusted switch rates. Adjusted regression models and PSM utilized listwise deletion for missing covariate data.

PSM is a statistical method designed to more accurately estimate differences in observational studies by balancing covariates (ie, reducing bias and confounding)^29,30^ and creating two similar (matched) samples. A logit propensity score (ie, conditional probability of being in a particular nicotine concentration policy region given observed covariates) was calculated using all covariates, and N.Am. smokers were then “matched” to UK smokers with similar characteristics (ie, the nearest propensity score value).^29^ Matching was conducted with replacement: a single N.Am. respondent could serve as the closest control for multiple UK respondents. Given the larger N.Am. sample, the matched sample included all UK respondents with valid covariate data and their matched N.Am. counterparts, excluding N.Am. respondents who were too dissimilar to any UK respondent. PSM was conducted separately at each follow-up, using respondents with valid data. To assess the validity of PSM, distributions of propensity scores in the two groups were assessed for balance (Supplementary Figures S2–S4 and Tables S1–S3).

In addition to statistically controlling for individual-level covariates, adjusted models were tested with a term capturing the year and quarter of assessment (eg, Q1 2019), to account for the potential effects of exogenous events during the study period (eg, e-cigarette or vaping product use-associated lung injury [EVALI], COVID-19 pandemic). The year-quarter × nicotine concentration policy region interaction term was also tested in an additional model to assess if the association between quarter and switching varied between N.Am. and UK.

To address the potential influence of factors not assessed in this study, we calculated an estimate of unmeasured confounding (the “*E*-value”); this represents the minimum effect of an unobserved confounder that would be necessary to fully attenuate the observed association of nicotine concentration policy region and switching (ie, explain away the association).^31,32^ Data were analyzed using Stata v.15.1.

## Results

### Participant Accrual

The analytic sample consisted of 10 082 smokers (N.Am.: *N* = 8835; UK: *N* = 1247), 25.1% retail and 74.9% online purchasers. The number of participants analyzed at each follow-up, with and without PSM, is displayed in Table 2.

**Table 2.** Association of Nicotine Concentration Policy Region and Switching in the Overall and Propensity Score Matched Samples

			Association of nicotine concentration policy region and past 30-day switching		
Follow-up assessment	Nicotine concentration policy region	Sample size *N* unadjusted (*N* adjusted)^a^	Unadjusted OR (95% CI)	Adjusted^b^ OR (95% CI)	*E*-Value^c^ unadjusted (adjusted)
Overall (unmatched) sample					
1-Month follow-up	North America	7487 (7241)	1.08 (0.91, 1.28)	1.24 (1.03, 1.49)	1.24 (1.47)
	United Kingdom	1078 (1034)	Ref.	Ref.	
3-Month follow-up	North America	6567 (6351)	1.30 (1.10, 1.53)	1.45 (1.21, 1.73)	1.54 (1.70)
	United Kingdom	863 (823)	Ref.	Ref.	
6-Month follow-up	North America	5527 (5340)	1.32 (1.10, 1.58)	1.63 (1.33, 1.98)	1.56 (1.87)
	United Kingdom	640 (608)	Ref.	Ref.	
Propensity score matched sample					
1-Month follow-up	North America	878	1.08 (0.85, 1.39)	1.10 (0.85, 1.43)	1.24 (1.28)
	United Kingdom	1032	Ref.	Ref.	
3-Month follow-up	North America	713	1.51 (1.20, 1.92)	1.59 (1.25, 2.02)	1.76 (1.83)
	United Kingdom	819	Ref.	Ref.	
6-Month follow-up	North America	524	1.68 (1.29, 2.18)	1.79 (1.37, 2.35)	1.92 (2.01)
	United Kingdom	608	Ref.	Ref.	

CI = confidence interval, OR = odds ratio.

^a^In the propensity score matched sample the unadjusted and adjusted models had identical sample sizes as there was no missing covariate data.

^b^Adjusted for all sociodemographic, smoking, and JUUL use characteristics.

^c^Minimum strength of association that an unmeasured confounder would need to have with both nicotine concentration policy region and switching, conditional on the measured covariates, to fully explain away the observed association.

### Baseline Characteristics

UK and N.Am. smokers who purchased JUUL significantly differed in all baseline sociodemographic and smoking characteristics (*p*s < 0.03; Table 1). N.Am. (vs. UK) smokers were significantly older, and a greater proportion were female, married, and White. On average, N.Am. (vs. UK) participants smoked more frequently, smoked more cigarettes per smoking day, and had higher levels of cigarette dependence; however, UK participants initiated smoking at a younger age. Perceived risks of JUUL use (vs. cigarette smoking) were lower in the UK than in N.Am.: 32.9% of UK respondents reported that JUUL was “much less harmful” than cigarettes, compared with 20.8% of N.Am. participants. A significantly greater proportion of UK participants reported using JUUL because: (1) it was advised by a physician; and (2) it “is healthier than cigarettes,” but a significantly smaller proportion of UK smokers reported using JUUL to “help quit smoking.”

Across all three follow-ups, over 82% of participants in both N.Am. and the UK reported using the highest nicotine concentration available (59 vs. 18 mg/mL). At each follow-up, a significantly greater proportion of N.Am. smokers reported using the highest nicotine concentration available (*p*s < 0.01; Table 1).

### Association of Nicotine Concentration Policy Region and Switching in the Unmatched Sample

**Figure 1.**
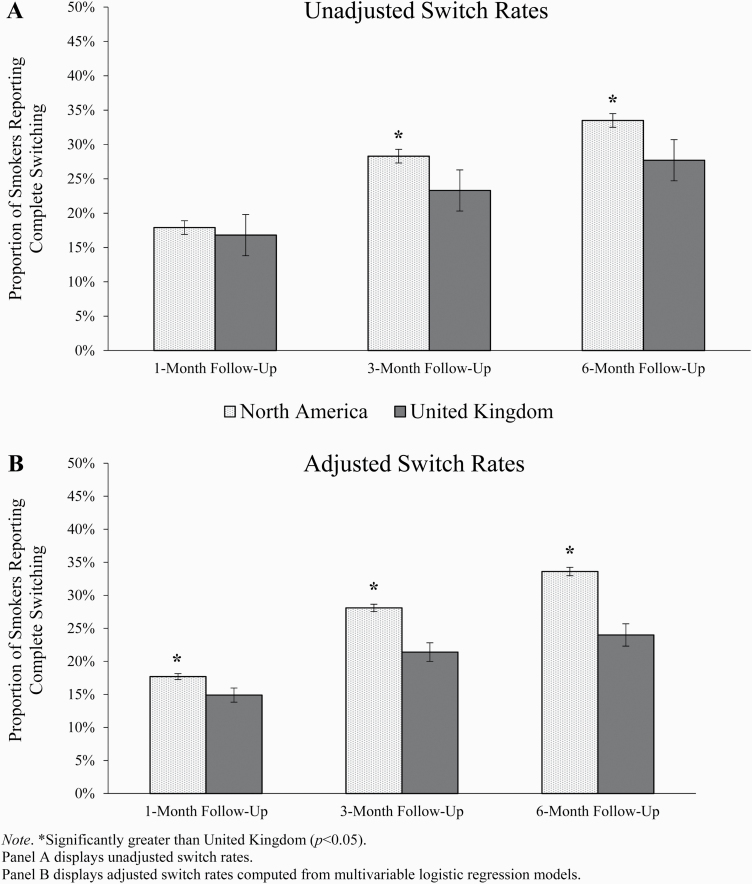
Proportion of North American and UK smokers reporting complete switching at 1-, 3-, and 6-month follow-up assessments in overall (unmatched) sample (±SE). *Significantly greater than United Kingdom (*p* < .05). Panel A displays unadjusted switch rates. Panel B displays adjusted switch rates computed from multivariable logistic regression models.

In the overall (unmatched) sample, without covariate adjustment, 17.9% of N.Am. smokers reported switching versus 16.8% of UK smokers at 1-month follow-up (difference [95% confidence interval, CI] = 1.1 [−1.3, 3.5]; Figure 1). At 3 months, the switching rates were 28.3% in N.Am. and 23.3% in the UK (difference [95% CI] = 5.0 [1.8, 8.1]), and at 6 months, 33.5% in N.Am. and 27.7% in the UK (difference [95% CI] = 5.8 [2.0, 9.7]). Unadjusted odds of switching were 30%–32% higher in N.Am. smokers (Table 2).

After covariate adjustment in logistic regression, switch rates were significantly greater in N.Am. (vs. UK) at all three timepoints, with odds of switching being 24%–63% higher among N.Am. (vs. UK) smokers (Table 2). The *E*-values indicate that for a confounder to fully explain the observed adjusted associations, its relation to switching and nicotine concentration policy region (odds ratio) would need to be at least 1.47.

Adjustment for calendar quarter had little effect: at each follow-up, parameter estimates for the association of nicotine concentration policy region and switching changed by less than 1% with the addition of the year-quarter term. The year-quarter × nicotine concentration policy region interaction term was significant at 1- and 3-month follow-ups (*p*s < 0.02).

The associations of each individual covariate and switching at each follow-up are displayed in Supplementary Table S4.

### Association of Nicotine Concentration Policy Region and Switching in the Matched Sample

The distribution of propensity scores in the N.Am. and UK samples following matching (ie, overlap in the propensity scores) suggest that PSM: (1) effectively balanced the samples across observed factors; (2) met the “common support” assumption; and (3) reduced underlying variation at all three follow-ups (Supplementary Figures S2–S4). Similarly, PSM significantly reduced differences in baseline sociodemographic and smoking-related characteristics between N.Am. and UK smokers (Supplementary Tables S1–S3).

**Figure 2.**
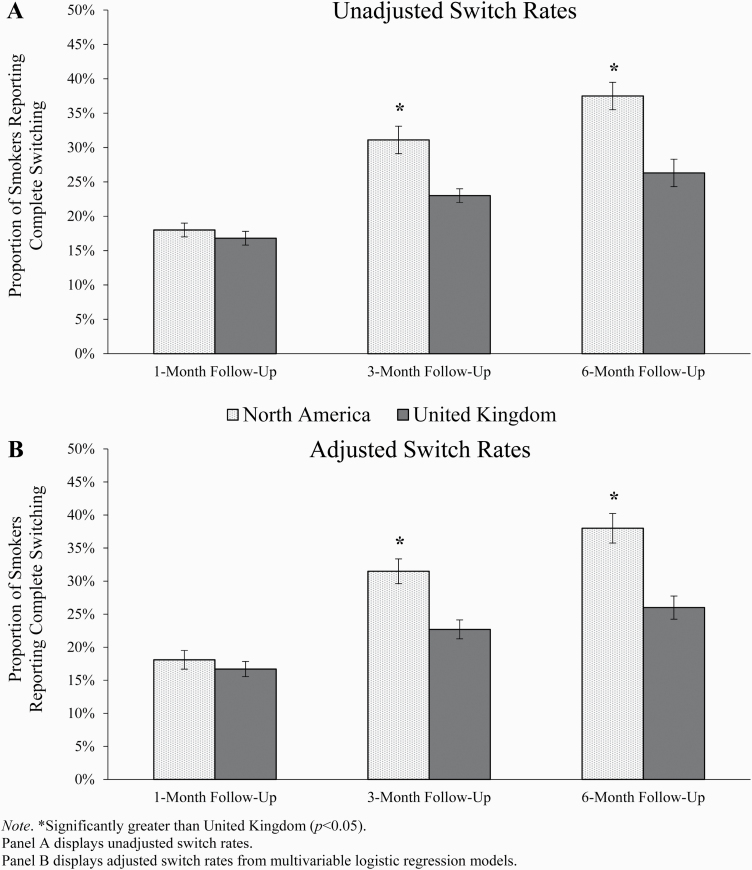
Proportion of North American and UK smokers reporting complete switching at 1-, 3-, and 6-month follow-up assessments in propensity score matched sample (±SE). *Significantly greater than United Kingdom (*p* < .05). Panel A displays unadjusted switch rates. Panel B displays adjusted switch rates from multivariable logistic regression models.

In the matched sample, without further covariate adjustment, 18.0% of N.Am. smokers reported switching vs. 16.8% of UK smokers at 1 month (difference [95% CI] = 1.2 [−2.4, 4.8]); these differences were not statistically significant (Figure 2). At 3 months, the switching rates were 31.1% in N.Am. and 23.0% in the UK (difference [95% CI] = 8.1 [3.5, 12.8]), and at 6 months switching rates were 37.5% in N.Am. and 26.3% in the UK (difference [95% CI] = 11.2 [5.5, 16.9]; Table 2). The unadjusted odds of switching were 51% (3 months) and 68% (6 months) higher among N.Am. (vs. UK) smokers.

A similar pattern of results was observed when logistic regression models additionally adjusted for baseline covariates: odds of switching among N.Am. (vs. UK) smokers increased to 59% and 79% at 3 and 6 months, respectively (Table 2). The *E*-values indicate that for a confounder to fully explain the observed associations, its relation to switching and nicotine concentration policy region (ie, odds ratio) would need to be at least 1.28.

As in the unmatched sample, parameter estimates for the association of nicotine concentration policy region and switching changed only slightly (1%–2%) with the addition of the year-quarter term. The year-quarter × nicotine concentration policy region interaction term was not significant at any follow-up (*p*s > 0.10).

### JUUL Use Across Follow-up

Prevalence of past 30-day JUUL use declined from 99% at 1 month to 80% at 6 months. The vast majority of participants who did not switch to exclusive JUUL use were dual users (vs. exclusive smokers or nonusers of both JUUL and cigarettes; Supplementary Table S5).

## Discussion

In this longitudinal study of adult smokers who purchased JUUL, rates of past 30-day switching 3 and 6 months following initial purchase were significantly lower in the UK, where nicotine concentrations are limited to 20 mg/mL, than in N.Am., where smokers were using higher nicotine levels in their JUUL devices. These differences in switch rates were evident in the unadjusted switching rates, and became more pronounced after statistical adjustment for relevant individual differences between N.Am. and UK smokers using four analytic approaches: (1) full (unmatched) sample without covariate adjustment; (2) unmatched sample with covariate adjustment; (3) PSM without covariate adjustment; and (4) PSM with additional covariate adjustment. The conclusions from all four models were consistent, demonstrating the robustness of the findings.

The approach that went furthest in accounting for differences between N.Am. and UK smokers utilized PSM to create UK and N.Am. samples that were similar in characteristics relevant to switching, and also included additional statistical adjustment for residual confounding in logistic regression models. In this model, N.Am. smokers’ relative odds of switching were 79% greater than those in the UK at 6 months. This difference in switching rates is of similar magnitude as the effect of using nicotine gum (vs. placebo) to assist smoking cessation.^2,33^ Given the health hazards associated with smoking,^34^ on a population level, differences of this magnitude could have critical implications for public health if past 30-day switching translates into increased rates of long-term sustained switching away from smoking.

As observed in previous studies of US smokers who purchased JUUL, switching rates increased over the 6-month follow-up period.^12,13^ Importantly, there was no intervention in the current study: participants purchased JUUL products on their own and received no instructions or advice regarding smoking. Although the switch rates observed in this observational study are not directly comparable to cessation trials, temporal patterns of increased switching over time stand in contrast to the pattern seen in traditional smoking-cessation trials, where rates of abstinence, including point prevalence abstinence, decline steeply over time.^2^ Consistent with other studies of switching among JUUL purchasers,^12,13^ participants generally continued use of JUUL across the study period; this pattern was also observed in a trial of ENDS for smoking cessation in the UK and is critical for noncombustible products intended as substitutes for cigarettes.^10^

There were numerous differences between smokers who purchased JUUL in the UK and those in N.Am. On average, N.Am. JUUL purchasers were older, heavier, and more dependent smokers, and a smaller proportion believed that JUUL was less harmful than smoking. Based on literature, this suggests that N.Am. (vs. UK) smokers generally had characteristics that would make them *less* likely to switch away from smoking.^21–26^ This made it even more striking that the N.Am. cohort had significantly higher switching rates at the 3- and 6-month follow-ups, before and after statistical adjustment, and suggests the higher nicotine concentrations used by N.Am. smokers may facilitate switching. The effect of PSM was to exclude smokers with the lowest likelihood of switching from the N.Am. sample, as these smokers had no close matches in the UK sample. Similarly, statistical adjustment for these differences increased rather than narrowed the differences in switching that were already evident in the unadjusted data.

These findings may inform the impact of the TPD’s limit on nicotine levels permitted in the UK and EU. The TPD was designed with the explicitly stated intention of equating nicotine delivery from ENDS to that of combustible cigarettes.^19^ Yet, evidence suggests that the 20 mg/mL limit on nicotine in ENDS does not consistently achieve this goal.^35,36^ In clinical pharmacokinetic studies of JUUL-naïve adult smokers, 59 mg/mL JUULpods deliver only approximately 50% of the nicotine delivered by cigarettes and reach only half the peak nicotine levels (*C*_max_).^37,38^ TPD-compliant JUULpods (18 mg/mL) deliver only approximately 20% of the nicotine delivered by cigarettes with a proportionately lower *C*_max_,^39,40^ and, concomitantly, have lower abuse liability than 59 mg/mL product.^40^ ENDS with higher nicotine concentrations may pose increased risk of dependence in nonsmokers, however a recent analysis of smokers who purchased JUUL in N.Am. (59 and 35 mg/mL concentrations) found that dependence on JUUL is lower than dependence on cigarettes, and that dependence decreases as smokers switch from smoking to exclusive JUUL use.^41^ Regulation based on actual nicotine *delivery*, rather than nicotine *concentration*, may be more appropriate to advance the goals of tobacco harm reduction.^15^

The findings of this study support the hypothesis that the higher nicotine concentrations used by N.Am. smokers, primarily 59 mg/mL, may contribute to the higher switching rates observed in N.Am. as compared with the UK. This result is consistent with experimental data that suggests that the substitutability of ENDS for combustible cigarettes increases with nicotine concentration.^42^ Aside from nicotine concentration, nicotine delivery of ENDS is also affected by factors including user behavior, battery power, and coil temperature.^15,43,44^ Since all participants in the present analyses were users of JUUL, a closed-system ENDS that does not permit adjustment of power or coil temperature, these and other device factors were held constant—hence variations by region likely reflect differences in nicotine concentration and delivery. In contrast, open system ENDS products are much more varied, and are capable of delivering greater levels of nicotine with lower nicotine concentrations^43,44^; although recent evidence suggests some may deliver very little nicotine.^45^ Accordingly, the results of this study may not generalize to open systems.

It is important to recognize that the data and analyses presented herein relate to smokers in each nicotine concentration policy region who, on their own initiative, purchased JUUL. This may be a substantially different population of smokers than seen in population-based analyses of ENDS users that define “users” as anyone who had even one puff on an ENDS in the past 30 days.^46–48^ It is not known what factors may affect the transition from trial to purchase and adoption, which could be influenced by product characteristics, individual differences, or by larger social, policy, and environmental factors. It is possible that such factors also contributed to the observed differences in outcomes between purchasers in the UK and in N.Am. Indeed, differences in nicotine concentration could have shaped differential adoption of JUUL in the UK and N.Am. over time. For example, if heavier, more dependent smokers considered or experienced the lower nicotine concentrations less satisfying (by reputation or experience), they might be less likely to buy JUUL in the UK. Since our sample consisted only of JUUL purchasers, the current data do not help disentangle such relationships or characterize the nature of this potential selection effect. However, the results can be interpreted as quantifying differences among smokers who chose to purchase similar products in each region.

While we were able to statistically adjust for differences in individual profiles of smokers who adopted JUUL in the UK and N.Am., comparisons across countries are confounded by other measured and unmeasured factors. Not only might there be other unmeasured, and therefore unadjusted, differences at the individual level, but there are also cultural, policy, and other societal-level variables that are not amenable to such statistical adjustments.

Several of the identifiable differences across nicotine concentration policy region favor greater switching success with ENDS in the UK: notably, the UK government and health authorities have been much more supportive of the use of ENDS use for moving away from smoking, to the point where the UK National Health Service suggests use of ENDS as an aid to cessation, and some centers even subsidize the cost of ENDS for smokers trying to quit.^49^ Some of these differences may have been captured in individual-level indicators such as smokers’ beliefs about the relative risks of ENDS, or their citing a doctor’s recommendation as a reason they bought a JUUL. Other differences would not be captured in these individual-level data. For example, the price of JUUL relative to cigarettes could have affected use of JUUL and switching. However, differences in price were minimal and JUUL, like other ENDS, was priced higher than cigarettes in both nicotine concentration policy regions.^50^ Other differences in ENDS policy or social environments, or more distal factors such as norms about using nicotine products, or even more general cultural differences, would not be captured in the covariates used in these analyses.

Strengths of the study include the longitudinal design, parallel samples of smokers who purchased the same ENDS device (JUUL) in both N.Am. and the UK, and use of several statistical techniques to adjust for differences between smokers in each region. The samples were also recruited and assessed using the same methods.

### Limitations

The greatest limitation, as discussed, is that there are many potential differences between UK and N.Am. smokers and in their milieu that could have influenced switching rates. Although we adjusted for key individual differences, the *E*-values suggest that unmeasured factors could still confound the associations observed herein. Additionally, there was no comparison group of smokers who did not purchase JUUL, so it is unknown whether switching would have occurred without JUUL—however, this lack of a control does not affect the between-region comparison of switching. As in similar observational studies, data are based on self-reports without biochemical verification of switching.^46–48^ Initial response rates are unknown, but to bias the findings rates would have to differ across regions. Also, use of other nicotine/tobacco products was not assessed. Future observational studies that assess sustained switching over longer periods of time and randomized trials in which nicotine concentration is experimentally manipulated are needed to determine if the identified association is causal.

## Conclusions

In this longitudinal study of N.Am. and UK smokers who purchased JUUL, switch rates were higher in the N.Am. users, where smokers were using higher nicotine concentrations. The results were robust to multiple adjustments for differences between the two nicotine concentration policy regions across different statistical approaches, including PSM on relevant observable characteristics. These results have implications for regulatory policy, as the availability of ENDS with nicotine concentrations greater than 20 mg/mL may facilitate switching away from cigarette among adult smokers.

## Supplementary Material

A Contributorship Form detailing each author’s specific involvement with this content, as well as any supplementary data, are available online at https://academic.oup.com/ntr.

ntab062_suppl_Supplementary_Material

ntab062_suppl_Supplementary_Taxonomy_Form
