# Supplementary material for: Differences in Switching Away From Smoking Among Adult Smokers Using JUUL Products in Regions With Different Maximum Nicotine Concentrations: North America and the United Kingdom
Source: Nicotine Tob Res. 2021 May 18;23(11):1821–30. doi: 10.1093/ntr/ntab062 (PMC8653762; doi:10.1093/ntr/ntab062)
Supplement: ntab062_suppl_Supplementary_Material [file ntab062_suppl_Supplementary_Material.pdf]

Differences in Rates of Switching Away from Smoking among Adult Smokers using JUUL System Products in Nicotine Concentration Policy Regions with Different Maximum Nicotine Concentrations: North America and the United Kingdom

ONLINE SUPPLEMENTAL MATERIAL

Figure S1. Participant Accrual and Flow Diagram

Figure S2. Propensity Score Distribution of Baseline Characteristics, Respondents at 1-Month Follow-Up

Table S3. Propensity Score Distribution of Baseline Characteristics, Respondents at 3-Month Follow-Up

Figure S4. Propensity Score Distribution of Baseline Characteristics, Respondents at 6-Month Follow-Up

Table S1. Summary Statistics for the Propensity-Score Adjusted Sample at 1-Month Follow-up

Table S2. Summary Statistics for the Propensity-Score Adjusted Sample at 3-Month Follow-up

Table S3. Summary Statistics for the Propensity-Score Adjusted Sample at 6-Month Follow-up

Table S4. Fully-Adjusted Logistic Regression Models in the Unmatched and Propensity Score Matched Samples

Table S5. Past 30-Day JUUL Use and Smoking at Each Follow-Up in North America and UK

Differences in Rates of Switching Away from Smoking among Adult Smokers using JUUL System Products in Nicotine Concentration Policy Regions with Different Maximum Nicotine Concentrations: North America and the United Kingdom

Figure S1. Participant Accrual and Flow Diagram

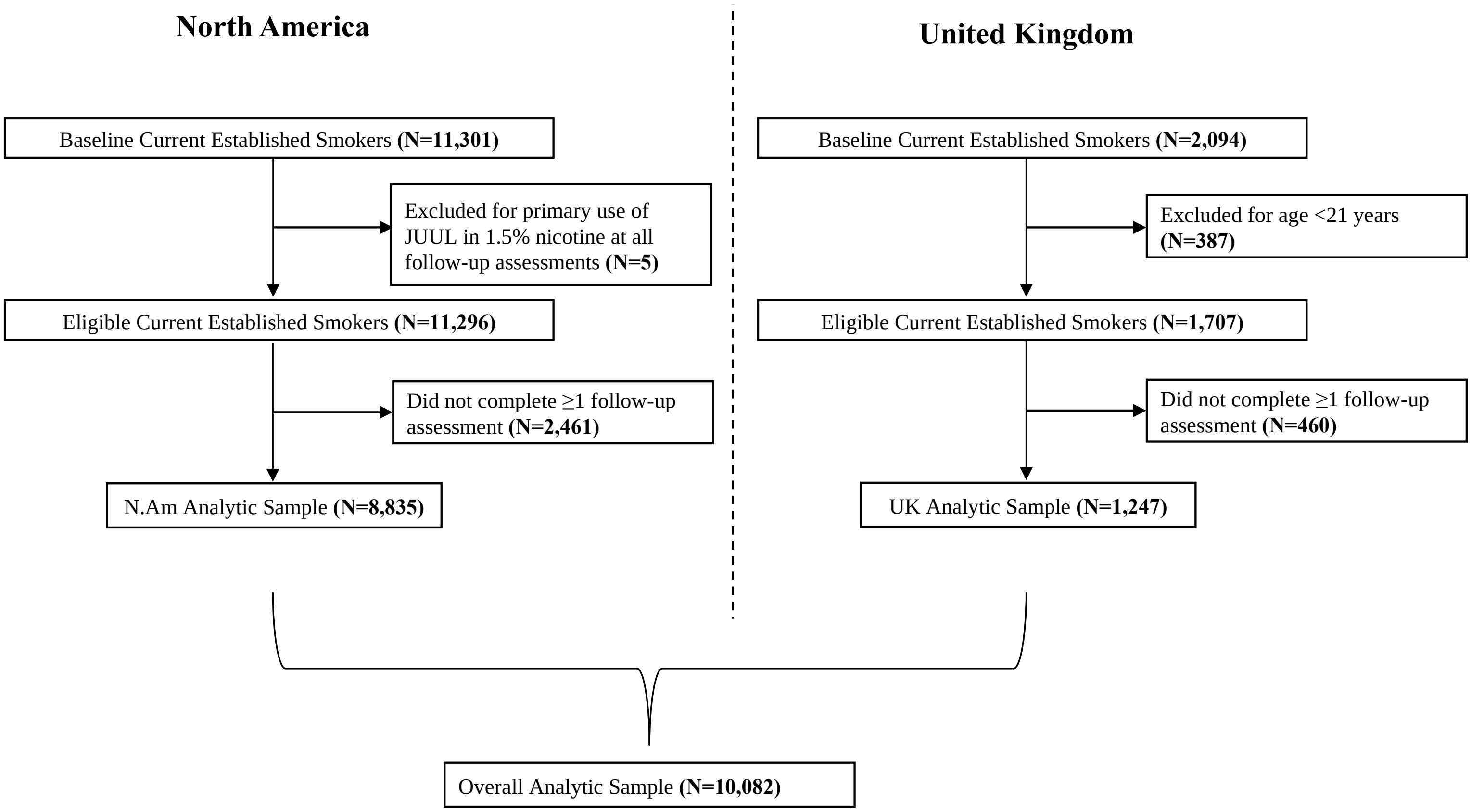

Differences in Rates of Switching Away from Smoking among Adult Smokers using JUUL System Products in Nicotine Concentration Policy Regions with Different Maximum Nicotine Concentrations: North America and the United Kingdom

Figure S2. Propensity Score Distribution of Baseline Characteristics, Respondents at 1-Month Follow-Up

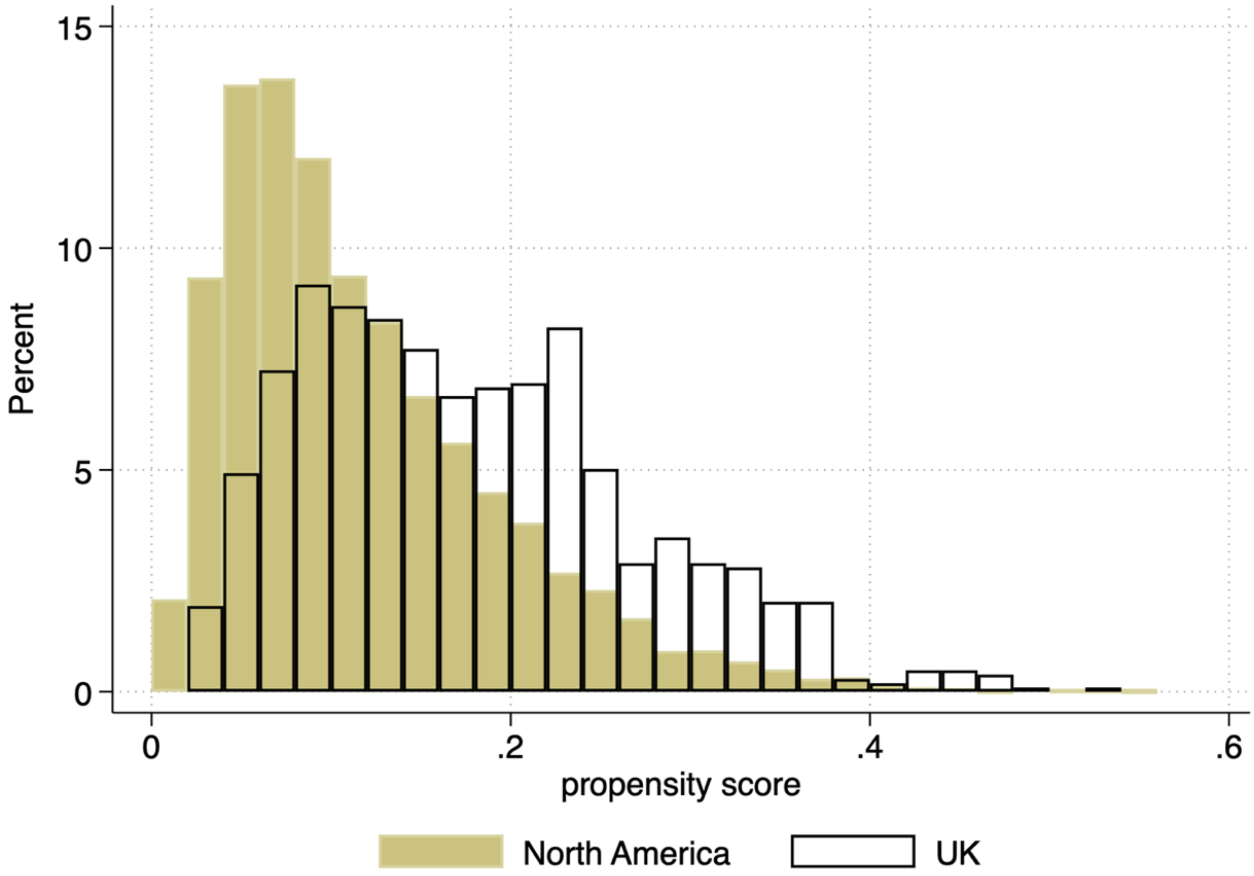

Figure S3. Propensity Score Distribution of Baseline Characteristics, Respondents at 3-Month Follow-Up

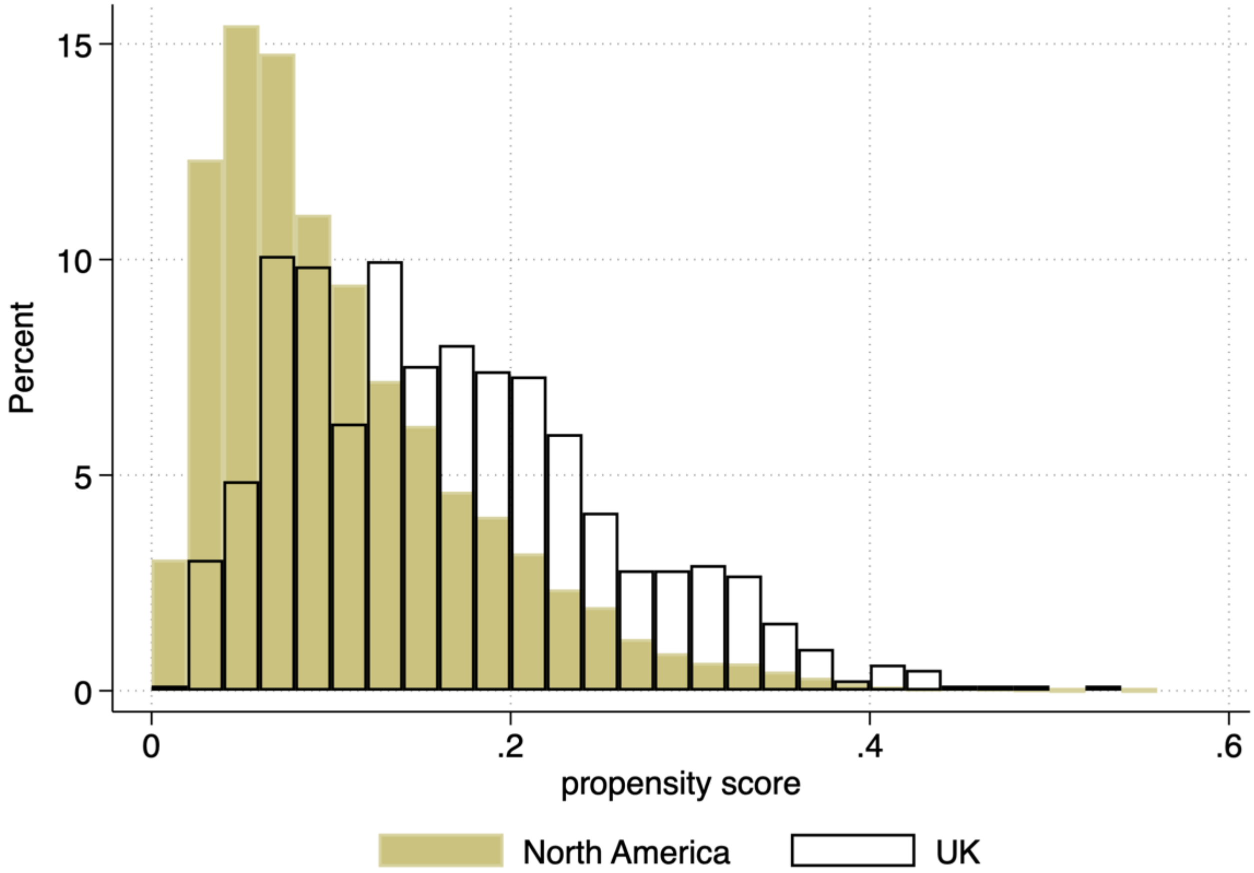

Differences in Rates of Switching Away from Smoking among Adult Smokers using JUUL System Products in Nicotine Concentration Policy Regions with Different Maximum Nicotine Concentrations: North America and the United Kingdom

Figure S4. Propensity Score Distribution of Baseline Characteristics, Respondents at 6-Month Follow-Up

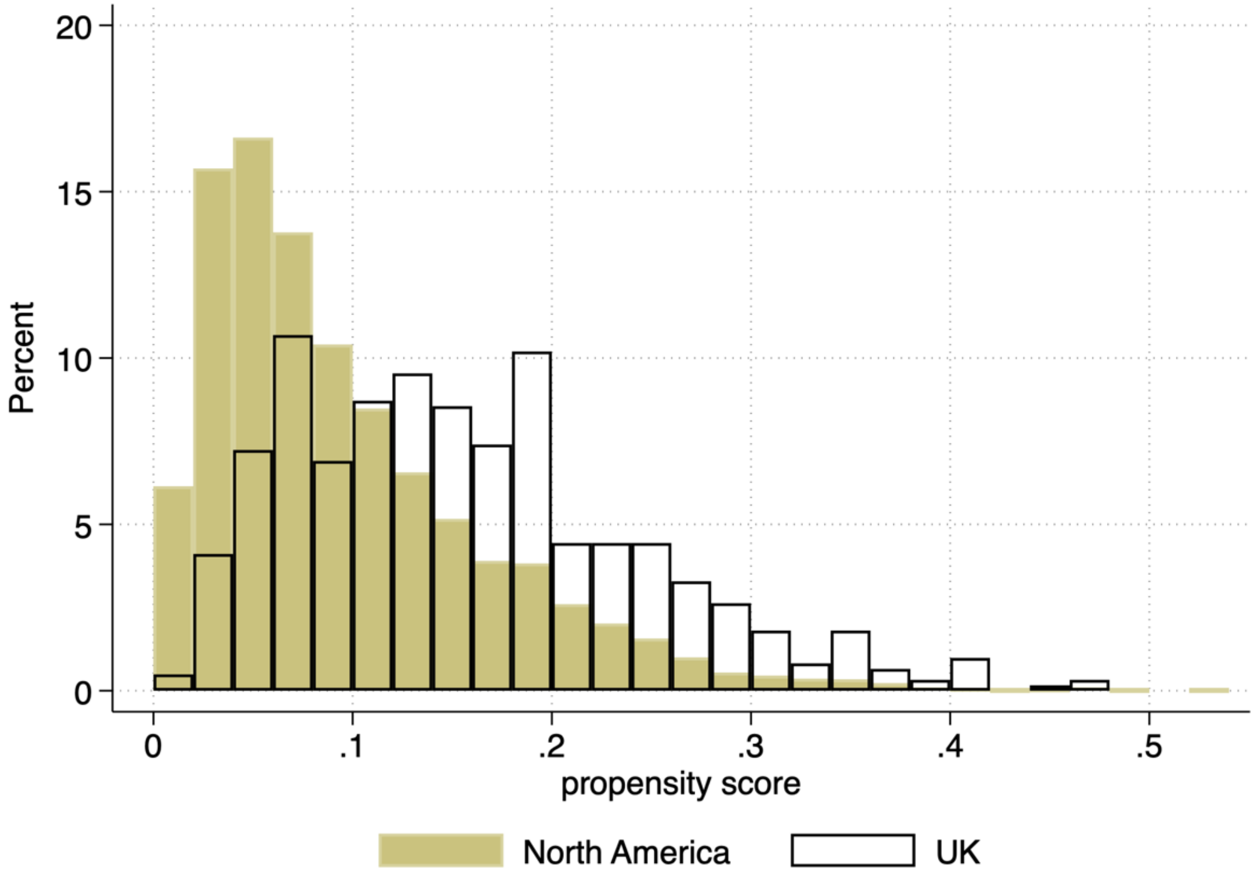

Differences in Rates of Switching Away from Smoking among Adult Smokers using JUUL System Products in Nicotine Concentration Policy Regions with Different Maximum Nicotine Concentrations: North America and the United Kingdom

Table S1. Summary Statistics for the Propensity-Score Adjusted Sample at 1-Month Follow-up

**Sociodemographic Characteristics**	North America Mean (*SD*) (N=878)	UK Mean (*SD*) (N=1,032)	Difference[a] (*p*-value)
Age	33.72 (0.34)	34.07 (0.36)	0.48
White-Race (Vs. Non-white)	0.82 (0.01)	0.80 (0.01)	0.31
Sex			
Male	0.66 (0.02)	0.65 (0.02)	0.89
Female	0.34 (0.02)	0.35 (0.02)	0.89
Transgender	0.00 (0.00)	0.00 (0.00)	1.00
Marital Status			
Married	0.24 (0.01)	0.25 (0.02)	0.66
Divorced, Separated, or Widowed	0.11 (0.01)	0.12 (0.01)	0.39
Never Married	0.65 (0.02)	0.63 (0.02)	0.33
**Smoking Characteristics**			
No. Cigarettes Smoked per Day	11.29 (0.32)	10.89 (0.33)	0.38
No. Days Smoked in Past 30 Days	23.51 (0.29)	23.67 (0.31)	0.71
Cigarette Dependence	2.98 (0.03)	2.98 (0.03)	0.99
Age started smoking regularly, yr	17.87 (0.10)	18.01 (0.13)	0.39
**JUUL Use Characteristics**			
Relative Harm of JUUL vs. Cigarettes			
Much less harmful	0.33 (0.02)	0.35 (0.02)	0.42
Less harmful	0.57 (0.02)	0.57 (0.02)	0.93
About the same level of harm	0.05 (0.01)	0.04 (0.01)	0.20
More harmful	0.00 (0.00)	0.00 (0.00)	—
Much more harmful	0.00 (0.00)	0.01 (0.00)	0.75
I don't know	0.05 (0.01)	0.04 (0.01)	0.61
Reason for JUUL Use			
Doctor advice	0.05 (0.01)	0.05 (0.01)	0.84
To help quit smoking	0.75 (0.01)	0.73 (0.02)	0.31
Less harmful than smoking	0.62 (0.02)	0.65 (0.02)	0.10

Note. The number of observations for each variable may be less than column heads due to missing data.
[a]Differences were tested with independent samples t-tests.

Differences in Rates of Switching Away from Smoking among Adult Smokers using JUUL System Products in Nicotine Concentration Policy Regions with Different Maximum Nicotine Concentrations: North America and the United Kingdom

Table S2. Summary Statistics for the Propensity-Score Adjusted Sample at 3-Month Follow-up

**Sociodemographic Characteristics**	North America Mean (*SD*) (N=713)	UK Mean (*SD*) (N=819)	Difference[a] (*p*-value)
Age	33.96 (0.39)	33.48 (0.37)	0.37
White-Race (Vs. Non-white)	0.84 (0.01)	0.82 (0.01)	0.57
Sex			
Male	0.68 (0.02)	0.68 (0.02)	0.80
Female	0.32 (0.02)	0.32 (0.02)	0.76
Transgender	0.00 (0.00)	0.00 (0.00)	0.58
Marital Status			
Married	0.25 (0.02)	0.24 (0.02)	0.54
Divorced, Separated, or Widowed	0.12 (0.01)	0.11 (0.01)	0.66
Never Married	0.63 (0.02)	0.65 (0.02)	0.40
**Smoking Characteristics**			
No. Cigarettes Smoked per Day	10.97 (0.37)	11.35 (0.38)	0.47
No. Days Smoked in Past 30 Days	23.68 (0.35)	23.47 (0.33)	0.66
Cigarette Dependence	2.95 (0.04)	2.98 (0.03)	0.60
Age started smoking regularly, yr	17.73 (0.12)	17.82 (0.12)	0.58
**JUUL Use Characteristics**			
Relative Harm of JUUL vs. Cigarettes			
Much less harmful	0.33 (0.02)	0.33 (0.02)	0.92
Less harmful	0.60 (0.02)	0.57 (0.02)	0.33
About the same level of harm	0.03 (0.01)	0.05 (0.01)	0.19
More harmful	0.00 (0.00)	0.00 (0.00)	0.33
Much more harmful	0.00 (0.00)	0.00 (0.00)	1.00
I don't know	0.04 (0.01)	0.05 (0.01)	0.49
Reason for JUUL Use			
Doctor advice	0.04 (0.01)	0.05 (0.01)	0.36
To help quit smoking	0.73 (0.02)	0.74 (0.02)	0.52
Less harmful than smoking	0.58 (0.02)	0.61 (0.02)	0.23

Note. The number of observations for each variable may be less than column heads due to missing data.
[a]Differences were tested with independent samples t-tests.

Differences in Rates of Switching Away from Smoking among Adult Smokers using JUUL System Products in Nicotine Concentration Policy Regions with Different Maximum Nicotine Concentrations: North America and the United Kingdom

Table S3. Summary Statistics for the Propensity-Score Adjusted Sample at 6-Month Follow-up

**Sociodemographic Characteristics**	North America Mean (*SD*) (N=524)	UK Mean (*SD*) (N=608)	Difference[a] (*p*-value)
Age	33.36 (0.43)	33.28 (0.42)	0.89
White-Race (Vs. Non-white)	0.81 (0.02)	0.82 (0.02)	0.72
Sex			
Male	0.70 (0.02)	0.68 (0.02)	0.44
Female	0.30 (0.02)	0.32 (0.02)	0.40
Transgender	0.00 (0.00)	0.00 (0.00)	0.58
Marital Status			
Married	0.21 (0.02)	0.23 (0.02)	0.43
Divorced, Separated, or Widowed	0.12 (0.01)	0.10 (0.01)	0.18
Never Married	0.66 (0.02)	0.67 (0.02)	0.86
**Smoking Characteristics**			
No. Cigarettes Smoked per Day	10.36 (0.34)	10.73 (0.40)	0.47
No. Days Smoked in Past 30 Days	23.09 (0.43)	22.89 (0.40)	0.74
Cigarette Dependence	2.92 (0.04)	2.96 (0.04)	0.42
Age started smoking regularly, yr	18.31 (0.16)	18.08 (0.15)	0.28
**JUUL Use Characteristics**			
Relative Harm of JUUL vs. Cigarettes			
Much less harmful	0.32 (0.02)	0.33 (0.02)	0.64
Less harmful	0.61 (0.02)	0.59 (0.02)	0.34
About the same level of harm	0.03 (0.01)	0.04 (0.01)	0.26
More harmful	0.00 (0.00)	0.00 (0.00)	—
Much more harmful	0.01 (0.00)	0.00 (0.00)	0.67
I don't know	0.04 (0.01)	0.05 (0.01)	0.69
Reason for JUUL Use			
Doctor advice	0.05 (0.01)	0.05 (0.01)	0.90
To help quit smoking	0.78 (0.02)	0.74 (0.02)	0.14
Less harmful than smoking	0.65 (0.02)	0.64 (0.02)	0.69

Note. The number of observations for each variable may be less than column heads due to missing data.
[a]Differences were tested with independent samples t-tests.

Table S4. Fully-Adjusted Logistic Regression Models Assessing Association of NCPR and Switching in the Unmatched and Propensity Score Matched Samples

Regressors	1-Month Follow-Up		3-Month Follow-Up		6-Month Follow-Up	
	Unmatched OR (*SD*)	PSM OR (*SD*)	Unmatched OR (*SD*)	PSM OR (*SD*)	Unmatched OR (*SD*)	PSM OR (*SD*)
North America vs. UK	1.24*	1.10	1.45**	1.59**	1.63**	1.79**
	(0.12)	(0.15)	(0.13)	(0.20)	(0.16)	(0.25)
Age, yr	0.98**	0.97**	0.98**	0.97**	0.98**	0.97**
	(0.00)	(0.01)	(0.00)	(0.01)	(0.00)	(0.01)
Female (vs. Male) Sex	0.88*	0.95	0.92	0.97	0.99	0.81
	(0.05)	(0.13)	(0.05)	(0.13)	(0.06)	(0.12)
Transgender (vs. Male) Sex	0.99	—	1.22	1.11	0.94	3.63
	(0.47)		(0.49)	(1.38)	(0.37)	(3.93)
Non-Hispanic White Race (vs. Other Race/Ethnicity)	0.97	1.03	0.91	1.02	1.06	1.10
	(0.07)	(0.17)	(0.06)	(0.17)	(0.07)	(0.20)
Divorced, Separated, or Widowed (vs. Married)	0.79*	0.50**	0.82*	0.96	0.83*	0.79
	(0.07)	(0.13)	(0.07)	(0.21)	(0.07)	(0.20)
Never Married (vs. Married)	0.71**	0.60**	0.69**	0.64**	0.68**	0.58**
	(0.05)	(0.10)	(0.05)	(0.10)	(0.05)	(0.11)
No. of Cigarettes Smoked per Smoking Day in Past 30 Days[a]	0.97**	0.97	0.99**	0.99	0.98**	0.99
	(0.00)	(0.02)	(0.00)	(0.01)	(0.00)	(0.01)
Age First Started Smoking Regularly	1.01	0.99	1.02**	1.01	1.02**	0.99
	(0.01)	(0.02)	(0.01)	(0.02)	(0.01)	(0.02)
Cigarette Dependence	0.88**	0.95	0.93*	0.92	0.90**	0.89
	(0.03)	(0.09)	(0.03)	(0.08)	(0.03)	(0.08)
Relative Harm of JUUL vs. Cigarettes						
Less harmful	0.79**	0.89	0.79**	0.83	0.82**	0.90
	(0.057)	(0.13)	(0.05)	(0.12)	(0.06)	(0.14)
About the same level of harm	0.53**	0.73	0.60**	0.61	0.541**	0.58
	(0.06)	(0.25)	(0.06)	(0.20)	(0.06)	(0.25)
More harmful	1.65	—	0.92	—	1.02	—
	(0.56)		(0.32)		(0.38)	
Much more harmful	1.28	3.01	1.55	0.59	0.73	0.31
	(0.52)	(2.45)	(0.61)	(0.83)	(0.32)	(0.41)
I don't know	0.64**	0.64	0.54**	0.41*	0.67**	0.96
	(0.09)	(0.22)	(0.07)	(0.15)	(0.08)	(0.33)
Reason for JUUL Use						
Advised by Doctor	1.24	1.32	1.35	1.73	1.58**	2.04*
	(0.23)	(0.41)	(0.21)	(0.56)	(0.25)	(0.61)
To Help Quit Smoking	0.88	0.81	1.10	1.07	1.08	0.78

Differences in Rates of Switching Away from Smoking among Adult Smokers using JUUL System Products in Nicotine Concentration Policy Regions with Different Maximum Nicotine Concentrations: North America and the United Kingdom

	(0.064)	(0.12)	(0.08)	(0.15)	(0.08)	(0.13)
Less Harmful Than Smoking	0.87*	0.85	0.98	0.80	0.96	1.03
	(0.06)	(0.12)	(0.06)	(0.11)	(0.06)	(0.16)
No. Observations	8,275	1,910	7,174	1,532	5,948	1,132
Psuedo R^2	0.04	0.04	0.03	0.03	0.03	0.04

Note. Abbreviations: NCPR, nicotine concentration policy region; OR, odds ratio; PSM. Propensity score matched; SD, standard deviation.

[a]Calculated as (number of days smoked cigarettes in the past 30 days × number of cigarettes smoked per day)/30.

$^{*}p<0.05$; $^{**}p<0.01$.

Differences in Rates of Switching Away from Smoking among Adult Smokers using JUUL System Products in Nicotine Concentration Policy Regions with Different Maximum Nicotine Concentrations: North America and the United Kingdom

Table S5. Past 30-Day Smoking and JUUL Use at Each Follow-Up in North America and UK

JUUL Use at Each Follow-Up	North America N (%)	UK N (%)
Overall (Unmatched) Sample		
**1-Month Follow-Up**		
Past 30-Day JUUL Use	7438 (99.4)	1067 (99.0)
Past 30-Day JUUL Use No Smoking	1331 (17.8)	180 (16.7)
Past 30-Day Dual Use[a]	6107 (81.6)	887 (82.3)
Past 30-Day Smoking No JUUL Use	40 (0.5)	10 (0.9)
No Past 30-Day Smoking or JUUL Use	9 (0.1)	1 (0.1)
**3-Month Follow-Up**		
Past 30-Day JUUL Use	6164 (93.8)	786 (91.1)
Past 30-Day JUUL Use No Smoking	1775 (27.0)	182 (21.1)
Past 30-Day Dual Use[a]	4389 (66.8)	604 (70.0)
Past 30-Day Smoking No JUUL Use	323 (4.9)	58 (6.7)
No Past 30-Day Smoking or JUUL Use	80 (1.2)	19 (2.2)
**6-Month Follow-Up**		
Past 30-Day JUUL Use	4800 (86.9)	509 (79.6)
Past 30-Day JUUL Use No Smoking	1652 (29.9)	136 (21.3)
Past 30-Day Dual Use[a]	3148 (57.0)	373 (58.3)
Past 30-Day Smoking No JUUL Use	529 (9.6)	90 (14.1)
No Past 30-Day Smoking or JUUL Use	198 (3.6)	41 (6.4)
Propensity Score Matched Sample[b]		
**1-Month Follow-Up**		
Past 30-Day JUUL Use	873 (99.4)	1021 (98.9)
Past 30-Day JUUL Use No Smoking	150 (17.1)	172 (16.7)
Past 30-Day Dual Use[a]	723 (82.3)	849 (82.3)
Past 30-Day Smoking No JUUL Use	3 (0.3)	10 (1.0)
No Past 30-Day Smoking or JUUL Use	2 (0.2)	1 (0.1)
**3-Month Follow-Up**		
Past 30-Day JUUL Use	676 (94.8)	747 (91.2)
Past 30-Day JUUL Use No Smoking	207 (29.0)	172 (21.0)
Past 30-Day Dual Use[a]	469 (65.8)	575 (70.2)
Past 30-Day Smoking No JUUL Use	27 (3.8)	55 (6.7)
No Past 30-Day Smoking or JUUL Use	10 (1.4)	17 (2.1)
**6-Month Follow-Up**		
Past 30-Day JUUL Use	466 (88.9)	486 (79.9)
Past 30-Day JUUL Use No Smoking	182 (34.7)	125 (20.6)
Past 30-Day Dual Use[a]	284 (54.2)	361 (59.4)
Past 30-Day Smoking No JUUL Use	43 (8.2)	87 (14.3)
No Past 30-Day Smoking or JUUL Use	15 (2.9)	35 (5.8)

Note. [a]Concurrent past 30-day use of JUUL and cigarette smoking.
[b]Frequency weights for matching with replacement were not applied to propensity score matched sample.
No tobacco products other than JUUL or cigarettes were included.
